# Fasting and cancer treatment in humans: A case series report

**DOI:** 10.18632/aging.100114

**Published:** 2009-12-31

**Authors:** Fernando M. Safdie, Tanya Dorff, David Quinn, Luigi Fontana, Min Wei, Changhan Lee, Pinchas Cohen, Valter D. Longo

**Affiliations:** ^1^ Andrus Gerontology Center and Department of Biological Sciences, University of Southern California, Los Angeles, CA 90089, USA; ^2^ University of Southern California Keck School of Medicine, Los Angeles, CA 90089, USA; ^3^ University of Southern California Norris Cancer Center, Los Angeles, CA 90089, USA; ^4^ Division of Geriatrics and Nutritional Science. Center for Human Nutrition, Washington University School of Medicine. Division of Nutrition and Aging. Istituto Superiore di Sanità, Rome, Italy; ^5^ UCLA Dept. of Pediatric Endocrinology, Los Angeles, CA 90095, USA; ^6^ These authors contributed equally to this work

**Keywords:** fasting, Cancer, Chemotherapy, Toxicity, Side-effect, IGF-I

## Abstract

Short-term fasting (48 hours) was shown to be effective in protecting normal cells and mice but not cancer cells against high dose chemotherapy, termed Differential Stress Resistance (DSR), but the feasibility and effect of fasting in cancer patients undergoing chemotherapy is unknown. Here we describe 10 cases in which patients diagnosed with a variety of malignancies had voluntarily fasted prior to (48-140 hours) and/or following (5-56 hours) chemotherapy. None of these patients, who received an average of 4 cycles of various chemotherapy drugs in combination with fasting, reported significant side effects caused by the fasting itself other than hunger and lightheadedness. Chemotherapy associated toxicity was graded according to the Common Terminology Criteria for Adverse Events (CTCAE) of the National Cancer Institute (NCI). The six patients who underwent chemotherapy with or without fasting reported a reduction in fatigue, weakness, and gastrointestinal side effects while fasting. In those patients whose cancer progression could be assessed, fasting did not prevent the chemotherapy-induced reduction of tumor volume or tumor markers. Although the 10 cases presented here suggest that fasting in combination with chemotherapy is feasible, safe, and has the potential to ameliorate side effects caused by chemotherapies, they are not meant to establish practice guidelines for patients undergoing chemotherapy. Only controlled-randomized clinical trials will determine the effect of fasting on clinical outcomes including quality of life and therapeutic index.

## Introduction

Chemotherapy can extend survival in patients diagnosed with a wide range of malignancies. However, side effects caused by toxicity to normal cells and tissues limit chemotherapy dose density and intensity, which may compromise efficacy. For instance, the cardiotoxicity and nephrotoxicity associated with the widely prescribed anti-cancer drugs, doxorubicin and cisplatin respectively limit their full therapeutic potential [1,4]. Thus, reduction of undesired toxicity by selective protection of normal cells without compromising the killing of malignant cells represents a promising strategy to enhance cancer treatment.

Calorie restriction (CR) is an effective and reproducible intervention for increasing life span, reducing oxidative damage, enhancing stress resistance and delaying/preventing aging and age-associated diseases such as cancer in various species, including mammals (mice, rats, and non- human primates) [5-8]. Recently, a fasting-based intervention capable of differentially protecting normal and cancer cells against high-dose chemotherapy in cell culture and in neuroblastoma-bearing mice was reported [9]. In the neuroblastoma xenograft model, mice were allowed to consume only water for 48 hours prior to etoposide treatment. Whereas high dose etoposide led to 50% lethality in *ad libitum* fed mice, fasting protected against the chemotoxicity without compromising the killing of neuroblastoma cells [9].

**Table 1.** Toxicity side effect survey. * Grade: 0 no symptom, 1 to 4 from mild, moderate, severe and life threatening (requires medical assistance) ** Fatigue: unusual tiredness which is not relieved by either a good night of sleep or rest. *** Weakness: lack of strength, vigor or firmness

**Toxicity Side Effect Survey**											
***General symptoms***	Grade*										
Fatigue **	0	1			2		3		4		
***4 Being extreme Fatigue***											
Weakness ***	0	1			2		3		4		
***4 Being Extreme Weakness***											
**Hair Loss**	0	1			2		3		4		
***4 Being Maximum Hair Loss***											
**Body Temperature**		**36.5°C /97.7°**	**37.0°C /98.6°**	**37.5°C /99.5°**	**38.0°C /100.4°**	**38.5°C /101.3°**	**39.0°C /102.2°**	**39.5°C /103.1°**	**40.0°C /104°**	**40.5°C /104.9°**	**41.0°C /105.8°**
**Head Aches**	0	1			2		3		4		
***4 Being the Worst Headache***											
**Gastrointestinal Side Effects**											
**Appetite**	0	1			2		3		4		
***4 Being Strong Appetite***											
**Nausea**	0	1			2		3		4		
***4 Being Unbearable Nausea***											
**Vomiting**	0	Mild			Moderate				Severe		
		< 2 times/Day			3-5 times/ Day				>5 times/Day		
**Diarrhea**	0	Mild			Moderate				Severe		
		< 2 times/Day			3-5 times/ Day				>5 times/Day		
**Abdominal Cramps**	0	1			2		3		4		
***4 Being Extreme Abdominal Cramps***											
**Mouth Sores**	0	1			2		3		4		
***4 Being Extremely Painful***											
**Dry Mouth**	0	1			2		3		4		
***4 Extreme Dryness***											
**CNS AND PNS Side Effects**											
**Short memory impairment**	0	1			2		3		4		
***4 Being High Impairment***											
**Numbness**	0	1			2		3		4		
***4 Being Maximum***											
**Tingling**	0	1			2		3		4		
***4 Being Maximum***											
**Neuropathy-motor**	0	1			2		3		4		
***4 Being = Paralysis***											

Previous human studies have shown that alternate day dietary restriction and short-term fasting (5 days) are well tolerated and safe [10-12]. In fact, children ranging from 6 months to 15 years of age were able to complete 14 to 40 hours of fasting in a clinical study carried out at the Children's hospital of Philadelphia [13]. Furthermore, alternate day calorie restriction caused clinical improvements and reduced markers of inflammation and oxidative stress in obese asthmatic patients [12,14].

Here, we report 10 cases of patients diagnosed with various types of cancer, who have voluntarily fasted prior to and following chemotherapy. The results presented here, which are based on self-assessed health outcomes (Table 1) and laboratory reports,  suggest that fasting is safe and raise the possibility that it can reduce chemotherapy-associated side effects. However, only a randomized controlled clinical trial can establish its efficacy.

**Figure 1.**
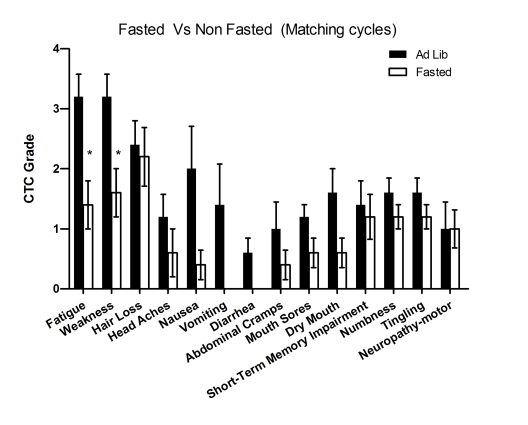
Self-reported side-effects after chemotherapy with or without fasting. Data represent average of CTCAE grade from matching fasting and non-fasting cycles (Ad Lib). 6 patients received either chemotherapy-alone or chemo-fasting treatments. Self-reported side effects from the closest two cycles were compared one another. Statistic analysis was performed only from matching cycles. Data presented as standard error of the mean (SEM). P value was calculated with unpaired, two tail t test. (*, P<0.05).

## Results

Ten cancer patients receiving chemotherapy, 7 females and 3 males with a median age of 61 years (range 44-78 yrs), are presented in this case series report. Four suffered from breast cancer, two from prostate cancer, and one each from ovarian, uterine, non small cell carcinoma of the lung, and esophageal adenocarcinoma. All patientsvoluntarily fasted for a total of 48 to 140 hours prior to and/or 5 to 56 hours following chemotherapy administered by their treating oncologists (Table 2, Table 3).

**Table 2.** Additional data from patients, including scheme of chemotherapy cycles, fasting regimens and tumor response. * also utilized low glycemic diet for 24 hours prior to fast. ** also utilized liquid diet for 24 hours after fast. n/a = not applicable, due to chemotherapy being administered in the adjuvant setting.

	**Cycle#**	**Fast(hours)**	**Chemotherapy**	**Tumor Response**
**Case 1**	1	140 pre 40 post	Docetaxel 75mg/m^2 ^**+** Cyclophosphamide 600mg/m^2^	n/a
	4	120 pre 24 post	Docetaxel 75mg/m^2 ^**+** Cyclophosphamide 600mg/m^2^	n/a
**Case 2**	4	72 pre 51 post	Docetaxel 64.6mg/m^2 ^+ carboplatin 485mg + 5FU 2415.7 mg/m^2^	---
	5	48 pre 56 post	Docetaxel 79 mg/m^2 ^+ carboplatin 470mg + 5FU 2415.7 mg/m^2^	Stable disease on CT/PET
	6	48 pre 56 post	Docetaxel 79 mg/m^2 ^+ carboplatin 470mg + 5FU 2415.7 mg/m^2^	Improvement on CT/PET. Refer to text.
	7	48 pre 56 post	Docetaxel 79 mg/m^2 ^+ carboplatin 470mg + 5FU 2415.7 mg/m^2^	---
	8	48 pre 56 post	Docetaxel 79 mg/m^2 ^+ carboplatin 470mg + 5FU 2415.7 mg/m^2^	Progression of Disease on CT/PET
**Case 3**	5- 12	60-66 pre 24 post	Docetaxel 75 mg/m^2^	See PSA Graph
**Case 4**	6	48 pre 24 post	Docetaxel 75mg/m^2 ^+ carboplatin 540mg	Stable disease CT/PET refer to text
**Case 5**	2	36 pre	Carboplatin 480 mg + Paclitaxel 280 mg	---
	3-4	60 pre	Carboplatin 480 mg + Paclitaxel 280 mg	87% decline in CA 125, Reduction in lymph nodes on CT
	5-6	60 pre 24post	Carboplatin 480 mg + Paclitaxel 280 mg	
**Case 6**	3	62 pre 24post	Gemcitabine 720 mg/m^2 ^(day1)+ GMZ 720 mg/m^2 ^Docetaxel 80 mg/m2 (Day8)	---
	4	62 pre 24post	Gemcitabine 720 mg/m^2 ^(day1)+ GMZ 720 mg/m^2 ^Docetaxel 80 mg/m2 (Day8)	---
	5-6	62 pre 24post	Gemcitabine 900 mg/m^2 ^(day1)+ GMZ 900 mg/m^2 ^Docetaxel 100 mg/m2 (Day8)	Stable disease on PET scan, No new MTS.
**Case 7**	1	65 pre 8 post	Docetaxel 60 mg/m^2^	See PSA Graph
	2-8	65 pre 25post*^	Docetaxel 75 mg/m^2^	See PSA Graph
**Case 8**	1-4	64 pre 24 post**	Docetaxel 75 mg/m^2 ^+ Cyclophosphamide 600 mg/m^2^	n/a
**Case 9**	1	48 pre	Doxorubicin 110 mg + Cyclophosphamide 1100 mg	n/a
	2-4	61 pre 4 post	Doxorubicin 110 mg + Cyclophosphamide 1100 mg	n/a
**Case 10**	1	60 pre	Docetaxel 75 mg/m^2 ^+ Carboplatin 400mg	n/a
	2	48 pre	Docetaxel 75 mg/m^2 ^+ carboplatin 400mg	n/a
	3	40 pre 24post	Docetaxel 75 mg/m^2 ^+ carboplatin 400mg	n/a
	4	48 pre 24post	Docetaxel 75 mg/m^2 ^+ carboplatin 400mg	n/a
	5	36 pre 24post	Docetaxel 75 mg/m^2 ^+ carboplatin 400mg	n/a
	6	20 pre 20post	Docetaxel 75 mg/m^2 ^+ carboplatin 400mg	n/a

**Table 3.** Additional demographical and clinical information of patients.

	**Gender**	**Age**	**Primary Neoplasia**	**Stage at Diagnosis**
**Case 1**	Female	51	Breast	IIA
**Case 2**	Male	68	Esophagus	IVB
**Case 3**	Male	74	Prostate	II
**Case 4**	Female	61	Lung (NSCLC)	IV
**Case 5**	Female	74	Uterus	IV
**Case 6**	Female	44	Ovary	IA
**Case 7**	Male	66	Prostate	IV/DI
**Case 8**	Female	51	Breast	IIA
**Case 9**	Female	48	Breast	IIA
**Case 10**	Female	78	Breast	IIA

### Case 1

**Figure 2.**
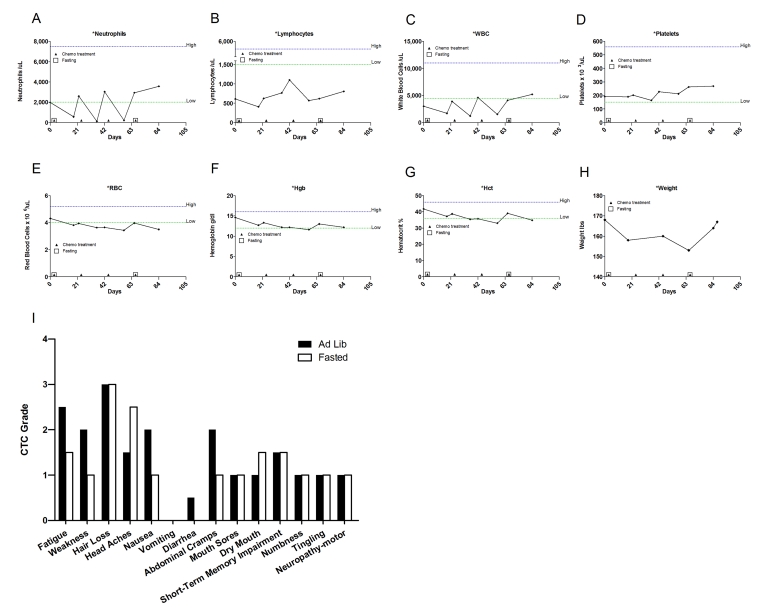
Laboratory values of blood cell counts for case 1. (**A**) Neutrophils; (**B**) Lymphocytes; (**C**) White blood cells, WBC; (**D**) Platelets; (**E**) Red blood cells, RBC (**F**) Hemoglobin, Hgb; (**G**) Hematocrit, Hct;  (**H**) Body weight. Filled triangle indicates day of chemotherapy; open square indicates fasting. Normal ranges of laboratory values are indicate by dash lines; (**I**) Self-reported side-effects after chemotherapy for case 1. Data represent the average of 2 cycles of chemo-alone *vs* the average of 2 cycles of chemo-fasting treatments.

This is a 51-year-old Caucasian woman diagnosed with stage IIA breast cancer receiving adjuvant chemo-therapy consisting of docetaxel (TAX) and cyclophosphamide(CTX). She fasted prior to her first chemotherapy administration. The fasting regimen consisted of a complete caloric deprivation for 140 hours prior and 40 hours after chemotherapy (180 hours total), during which she consumed only water and vitamins. The patient completed this prolonged fasting without major inconvenience and lost 7 pounds, which were recovered by the end of the treatment (Figure 2H). After the fasting-chemotherapy cycle, the patient experienced mild fatigue, dry mouth and hiccups (Figure 2I); nevertheless she was able to carry out her daily activities (working up to 12 hours a day). By contrast, in the subsequent second and third treatment, she  received chemotherapy accompanied by  a regular diet and complained of moderate to severe fatigue, weakness, nausea, abdominal cramps and diarrhea (Figure 2I). This time the side effects forced her to withdraw from her regular work schedule. For the forth cycle, she opted to fast again, although with a different regimen which consisted of fasting 120 hours prior to and 24 hours post chemotherapy. Notably, her self-reported side effects were lower despite the expected cumulative toxicity from previous cycles. Total white blood cell (WBC) and absolute neutrophil counts (ANC) were slightly better at nadir when chemotherapy was preceded by fasting (Figure 2A, C; Supplementary Table 1). Furthermore, platelets level decreased by 7-19% during cycles 2 and 3 (*ad libitum* diet) but did not drop during the first and forthcycles (fasting), (Figure 2D). After the forthchemotherapy cycle combined with 144-hour fast her ANC, WBC, and platelet counts reached their highest level since the start of chemotherapy 80 days earlier (Figure 2A, C and D).

### Case 2

**Figure 3.**
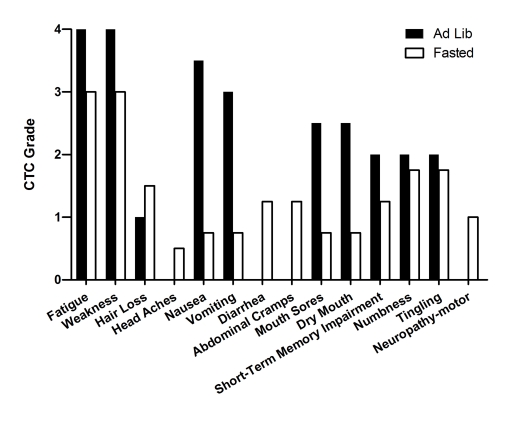
Self-reported side-effects after chemotherapy for case 2. Data represent the average of 3 cycles of chemo-alone *vs* the average of 5 cycles of chemo-fasting treatments.

This is a 68-year-old Caucasian male diagnosed in February 2008 with esophageal adenocarcinomametastasic to the left adrenal gland. The initial treatment consisted of 5-fluorouracil (5-FU) combined with cisplatin(CDDP) concurrent with radiation for the first two cycles. Throughout these first two cycles, the patient experienced multiple side effects including severe weakness, fatigue, mucositis, vomits and grade 2-3 peripheral neuropathy (Figure 3). During the third cycle, 5-FU administration was interrupted due to severe nausea and refractory vomiting (Figure 3). In spite of the aggressive approach with chemotherapy and radiation, his disease progressed with new metastases to the right adrenal gland, lung nodules, left sacrum, and coracoid process documented by computed tomography - positron emission tomography (CT-PET) performed in August 2008. These prompted a change in his chemotherapy regimen for the fourth cycle to carboplatin (CBDCA) in combination with TAX and 5-FU (96 hour infusion) (Table 2). During the fourth cycle, the patient incorporated a 72-hour fast prior to chemotherapy and continued the fast for 51 hours afterward, consuming only water. The rationale for the 51 hour post-chemotherapy fasting was to cover the period of continuous infusion of 5-FU. The patient lost approximately 7 pounds, of which 4 were regained during the first few days after resuming *ad libitum* diet (data not shown). Although a combination of three chemotherapeutic agents were used during this cycle, self-reported sideeffects were consistently less severe compared to cycles in which calories were consumed ad lib (Figure 3). Prior to his fifth cycle the patient opted to fast again. Instead of receiving the 5-FU infusion for 96 hours, as he did previously, the same dose of the drug was administered within 48 hours, and the fasting regimen was also modified to 48 hours prior and 56 hours post chemotherapy delivery. Self-reported side effects were again less severe than those in association with  *ad libitum* diet and the restaging CT-PET scan indicated objective tumor response, with decreased standard uptake values (SUV) in the esophageal mass,the adrenal gland metastases, and the lung nodule. From the sixth to eight cycle, the patient fasted prior to and following chemotherapy treatments (Table 2). Fasting was well tolerated in all cycles and chemotherapy-dependent side effects were reduced except for mild diarrhea and abdominal cramps that were developed during the seventh cycle (Figure 3). Ultimately, the patient's disease progressed and the patient died in February 2009.

### Case 3

**Figure 4.**
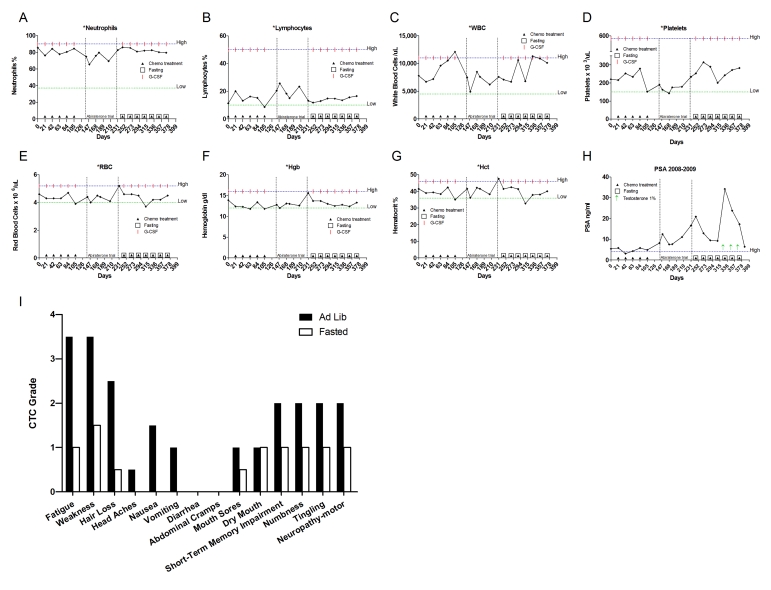
Laboratory values of blood cell counts for case 3. (**A**) Neutrophils; (**B**) Lymphocytes; (**C**) White blood cells, WBC; (**D**) Platelets; (**E**) Red blood cells, RBC (**F**) Hemoglobin, Hgb; (**G**) Hematocrit, Hct; (H) Prostate specific antigen (PSA) level. The patient was enrolled in abiraterone acetate (CYP17 inhibitor) trial for 90 days indicated by vertical dash lines. The patient also received G-CSF (Neulasta) on the day of chemotherapy except during the treatment with abiraterone acetate. Filled triangle indicates day of chemotherapy; open square indicates fasting, arrow indicates testosterone application (cream 1%). Normal ranges of laboratory values are indicated by horizontal dash lines; (**I**) Self-reported side-effects after chemotherapy for case 3. Data represent the average of 5 cycles of chemo-alone *vs* the average of 7 cycles of chemo-fasting treatments.

This is a 74-year-old Caucasian man who was diagnosed in July 2000 with stage II prostate adeno-carcinoma, Gleason score 7 and baseline PSA level of 5.8 ng/ml. He achieved an undetectable PSA nadir after radical prostatectomy performed in September of 2000, but experienced biochemical recurrence inJanuary 2003 when PSA rose to 1.4 ng/ml. Leuprolide acetate together with bicalutamide and finasteride were prescribed. However, administration of these drugs had to be suspended in April 2004 due to severe side effects related to testosterone deprivation. Additional therapies including triptorelin pamoate, nilutamide, thalidomide, CTX and ketoconazole failed to control the disease. In 2007 the patient's PSA level reached 9 ng/ml and new metastases were detected on bone scan. Despite that TAX at 25mg/m^2 ^was administered on weekly basis,  the PSA level continued to increase, reaching 40.6 ng/ml (data not shown).  Bevacizumab was added to the treatment and only then did the PSA drop significantly (data not shown). Throughout the cycles with chemotherapy the patient experienced significant side effects including fatigue, weakness, metallic taste, dizziness, forgetfulness, short-term memory impairment and peripheral neuropathy (Figure 4I). After discontinuing the chemotherapy, his PSA rose rapidly. TAX was resumed at 75mg/m^2 ^every 21 days, and was complemented with granulocytic colony stimulating factor (G-CSF). Once again the patient suffered significant side effects (Figure 4I). In June 2008, chemotherapy was halted. The patient was enrolled in a phase III clinical trial with abiraterone acetate, a drug that can selectively block CYP17, a microsomal enzyme thatcatalyzes a series of reactions critical to nongonadal androgenbiosynthesis [15]. During the trial, the patient's PSA levels increased to 20.9ng/dl (Figure 4H), prompting resumption of chemotherapy and G-CSF. This time the patient opted to fast prior to chemotherapy.  His fasting schedule consisted of 60 hours prior to and 24 post drug administration (Table 2). Upon restarting chemotherapy with fasting the PSA level dropped, and notably, the patient reported considerably lower side effects than in previous cycles in which he consumed calories ad-lib (Figure 4I). He also experienced reduced myelosuppression (Figure4A-G). During the last three cycles, in addition to fasting, the patient applied testosterone (cream, 1%) for five days prior to chemotherapy. As a consequence the PSA level along with the testosterone level increased dramatically. Nonetheless, 3 cycles of chemotherapy combined with fasting reduced PSA from 34.2 to 6.43 ng/ml (Figure 4H). These results imply that the cytotoxic activity of TAX to cancer cells was not blocked by fasting.

**Figure 5.**
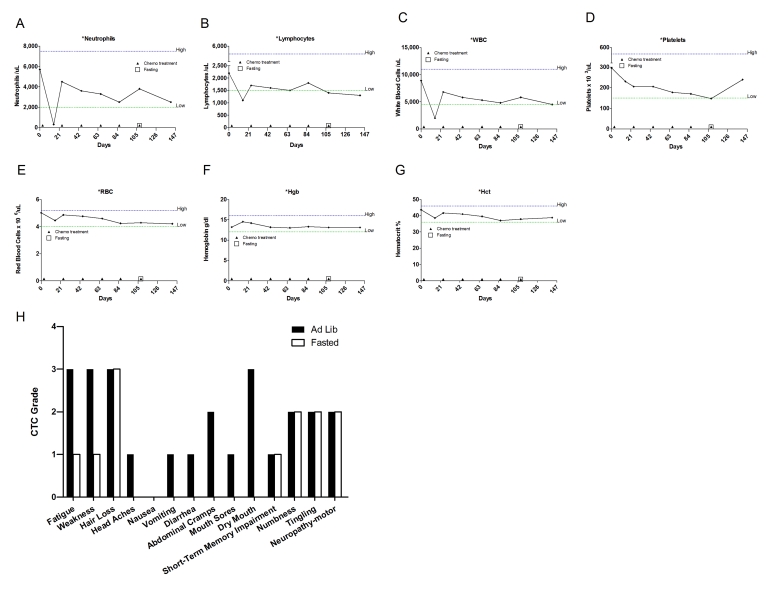
Laboratory values of blood cell counts for case 4. (**A**) Neutrophils; (**B**) Lymphocytes; (**C**) White blood cells, WBC; (**D**) Platelets; (**E**) Red blood cells, RBC (**F**) Hemoglobin, Hgb; (**G**) Hematocrit, Hct;   Filled triangle indicates day of chemotherapy; open square indicates fasting. Normal ranges of laboratory values are indicated by dash lines; (**H**) Self-reported side-effects after chemotherapy for case 4. Data represent the average of 5 cycles of chemo-alone *vs* 1 cycle of chemo-fasting treatment.

### Case 4

This is a 61-year-old Caucasian female who was diagnosed in June 2008 with poorly differentiated non-small cell lung carcinoma (NSCLC). A staging PET scan documented a hypermetabolic lung mass, multiple mediastinal and left perihilar lymph nodes, and widespread metastatic disease to the bones, liver, spleen, and pancreas. The initial treatment commenced with the administration of TAX 75 mg/m^2^ and CBDCA 540mg every 21 days. Although she was on a regular diet, during the first 5 cycles she lost an average of 4 pounds after each treatment, most likely due to chemotherapy-induced anorexia. The patient reported that she did return to her original weight but only after three weeks of the drug administration, just before a new cycle. Additional side effects included severe muscle spasms, peripheral neuropathy, significant fatigue, mucositis, easy bruising and bowel discomfort (Figure 5H). During the sixthcycle,which consisted of the same drugs and dosages, the patient fasted for 48-hours-prior and 24-hours-post chemotherapy. She lost approximately 6 pounds during the fasting period, which she recovered within 10 days (data not shown). Besides mild fatigue and weakness, the patient did not complain of any other side effect which was experienced during the five previous cycles (Figure 5H). Cumulative side effects such as peripheral neuropathy, hair loss and cognitive impairment were not reversed. By contrast self-reported acute toxic side effects were consistently reduced when chemotherapy was administered in association with fasting (Figure 5H). In the sixth and last cycle,  the patient reported that her strength returnedmore quickly after the chemotherapy so that she was able to walk 3 miles three days after the drug administration, whereas in previous cycles (*ad libitum* diet) she had experienced severe weakness and fatigue which limited any physical activity. No significant differences were observed in the patient's blood analysis (Figure 5A-G). The last PET scan performed on February 2009 showed stable disease in the main mass (lungs) and decreased uptake in the spleen and liver when compared to her baseline study.

**Figure 6.**
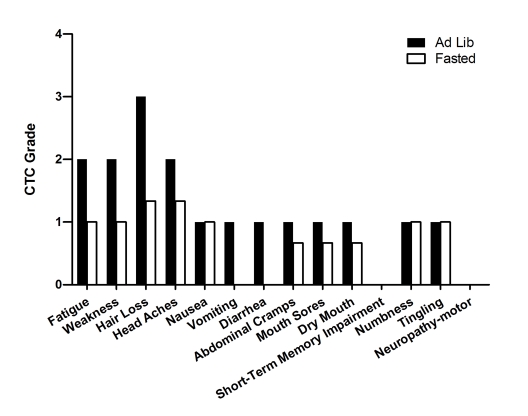
Self-reported side-effects after chemotherapy for case 5. Data represent 1 cycle of chemotherapy-alone (first cycle) *vs* the average of 5 cycles of chemo-fasting treatments.

### Case 5

This is a 74 year-old woman diagnosed in 2008 with stage IV uterine papillary serous carcinoma. Surgery (Total Abdominal Hysterectomy-Bilateral Salpingo-Oopherectomy, TAH-BSO, with lymph node dissection) followed by adjuvant chemotherapy were recommended. Due to significant enlargement of the right ureter, a right nephrectomy was also performed. Post-operatively, six cycles of CBDCA (480mg) and paclitaxel (280mg) were administered every 21-days. During the first treatment the patient maintained her regular diet and experienced fatigue, weakness, hair loss, headache and gastrointestinal discomfort (Figure 6). By contrast, during cycles 2-6, the patient fasted before and after chemotherapy, and reported a reduction in the severity of chemotherapy-associated side effects (Table 2; Figure 6). Fasting did not appear to interfere with chemotherapy efficacy, as indicated by the 87% reduction in the tumor marker CA-125 after the forthcycle (data not shown).

### Case 6

**Figure 7.**
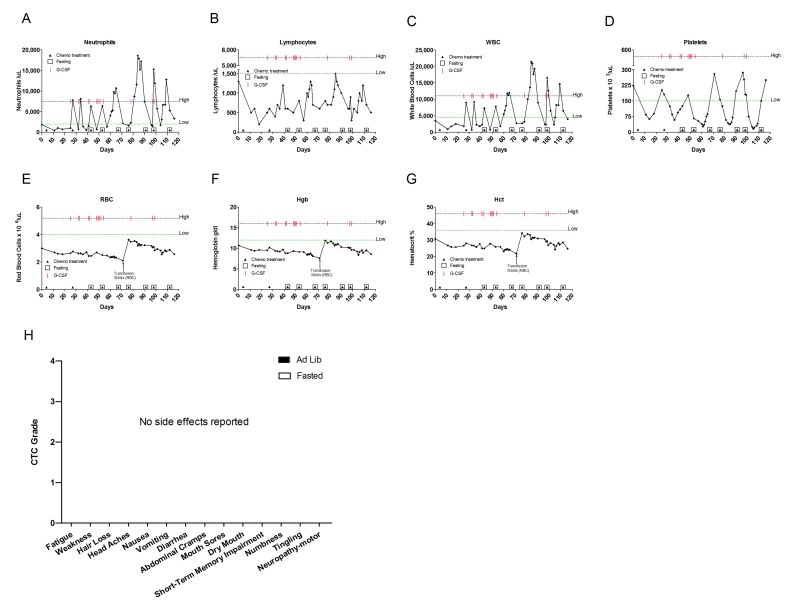
Laboratory values of blood cell counts for case 6. (**A**) Neutrophils; (**B**) Lymphocytes; (**C**) White blood cells, WBC; (**D**) Platelets; (**E)** Red blood cells, RBC (**F)** Hemoglobin, Hgb; (**G**) Hematocrit, Hct; Filled triangle indicates day of chemotherapy; open square indicates fasting. Normal ranges of laboratory values are indicated by dash lines. The patient received red blood cell transfusion (3 units) on day 71 and also received G-CSF (Neulasta) as indicated.

This is a 44-year-old Caucasian female diagnosed with a right ovarian mass (10x12 cm.) in July 2007. Surgery (TAH-BSO) revealed stage IA carcinosarcoma of the ovary with no lymph node involvement. Adjuvant treatment consisted of six cycles of ifosfamide and CDDP, administered from July to November of 2007. She remained free of disease until an MRI revealed multiple new pulmonary nodules in August 2008. Consequently chemotherapy with taxol, carboplatin and bevacizumab was initiated. By November, however, a CT scan showed progression of the cancer. Treatment was changed to gemcitabine plus TAX complemented with G-CSF (Neulasta) (Table 2 and Supplementary Table 2). After the first dose of gemcitabine (900 mg/m^2^), the patient experienced prolonged neutropenia (Figure 7A) and thrombocytopenia (Figure 7D), which forced a delay of day 8 dosing. During the second cycle the patient received a reduced dose of gemcitabine (720 mg/m^2^), but again developed prolonged neutropenia and thrombocytopenia, causing dose delays. For the third and subsequent cycles, the patient fasted for 62 hours prior to and 24 hours after chemotherapy. The patient not only did not find hardship on carrying out the fasting but also showed a faster recovery of her blood cell counts, allowing the completion of the chemotherapy regimen (gemcitabine 720mg/m^2^ on day 1 plus gemcitabine 720mg/m^2^ and TAX 80mg/m^2^ on day 8). During the fifth cycle, she fasted under the same regimen and received a full dose of gemcitabine (900mg/m^2^) and TAX (Table 2 and Supplementary Table 2). Her complete blood count showed consistent improvement during the cycles in which chemotherapy was combined with fasting. A trend in which nadirs were slightly less pronounced and the zeniths were considerably higher in ANC, lymphocyte and WBC counts was observed (Figure 7A, B, C, respectively; Supplementary Table 2). During the first and second cycle (*ad libitum* diet) gemcitabinealone induced prolonged thrombocytopenia, which took 11 and 12 days to recover, respectively (Figure 7D; Supplementary Table 2) but  following the first combined fasting-gemcitabine treatment (thirdand subsequent cycles), the duration of thrombocytopenia was significantly shorter (Figure 7D; Supplementary Table 2).

**Figure 8.**
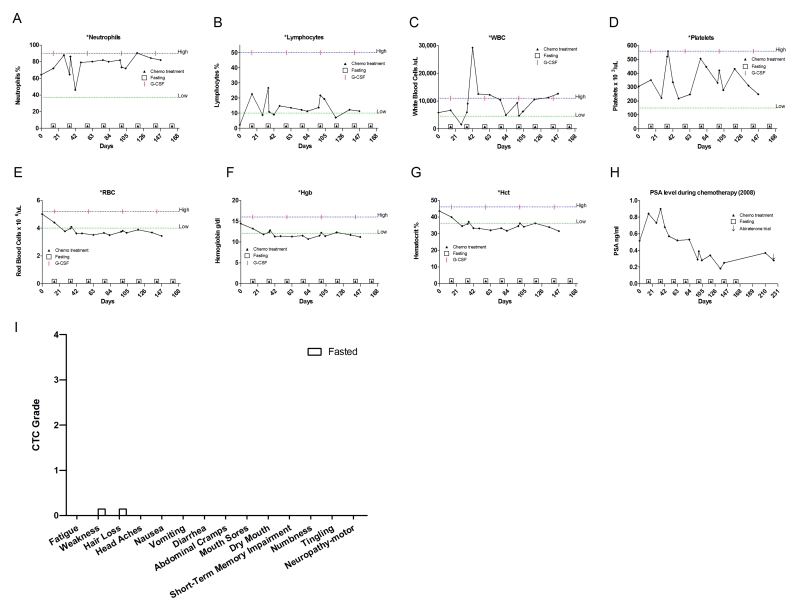
Laboratory values of blood cell counts for case 7. (**A**) Neutrophils; (**B**) Lymphocytes; (**C**) White blood cells, WBC; (**D**) Platelets; (**E**) Red blood cells, RBC (**F**) Hemoglobin, Hgb; (**G**) Hematocrit, Hct; (**H**) Prostate specific antigen (PSA) level. Filled triangle indicates day of chemotherapy; open square indicates fasting, arrow indicates abiraterone administration. Normal ranges of laboratory values are indicate by dash lines. The patient also received G-CSF (Neulasta) as indicated; (**I**) Self-reported side-effects after chemotherapy for case 7. Data represent the average of 8 cycles of chemo-fasting treatments.

### Case 7

This is a 66-year-old Caucasian male who was diagnosed in July 1998 with prostate adenocarcinoma, Gleason score 8. A Prosta Scint study performed in the same year displayed positive uptake of the radiotracer in the right iliac nodes, consistent with stage D1 disease. The patient was treated with leuprolide, bicalutamide and finasteride. In December 2000, the diseases progressed. He started on a second cycle with leuprolide acetate and also received High Dose Rate (HDR) brachytherapy and external beam radiation with Intensity Modulated Radiation Therapy (IMRT) to the prostate and pelvis. In April 2008, a Combidex scan revealed a 3 x 5 cm pelvic mass and left hydronephrosis prompting initiation of TAX chemotherapy supplemented with G-CSF.  The patient received 60-75 mg/m^2 ^of TAX for 8 cycles. Throughout this period the patient fasted for 60-66 prior to and 8-24 hours following chemotherapy(Table 2). Side effects from fasting included grade one lightheadedness (accordingly CTCAE 3.0) and a drop in blood pressure, none of which interfered with his routine. Chemotherapy-associated self-reported side effects included grade one sensory neuropathy (Figure 8I). The patient's ANC, WBC, platelet and lymphocyte levels remained in the normal range throughout treatment, although he did develop anemia (Figure8A-G). PSA levels consistently decreased, suggesting that fasting did not interfere with the therapeutic benefit of the chemo-treatment (Figure 8H).

**Figure 9.**
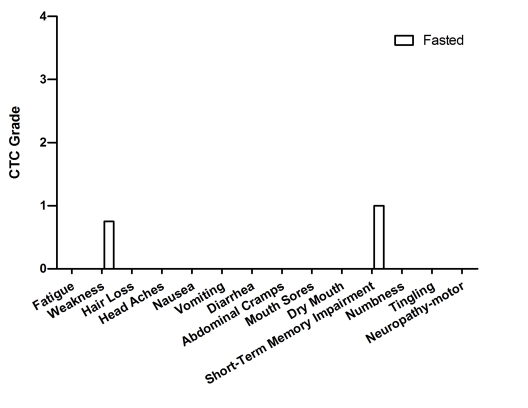
Self-reported side-effects after chemotherapy for case 8. Data represent the average of 4 cycles of chemo-fasting treatments.

### Case 8

This is a 53-year-old Caucasian female who was diagnosed with stage IIA breast cancer (HER2+) in 2008. After a lumpectomy procedure, she received 4 cycles of adjuvant chemotherapy with TAX (75mg/m^2^) and CTX (600mg/m^2^) every 21 days. For all 4 cycles the patient fasted 64 hours prior to and 24 hours post chemotherapy administration (Table 2). Self-reported side effects included mild weakness and short-term memory impairment (Figure 9).

### Case 9

**Figure 10.**
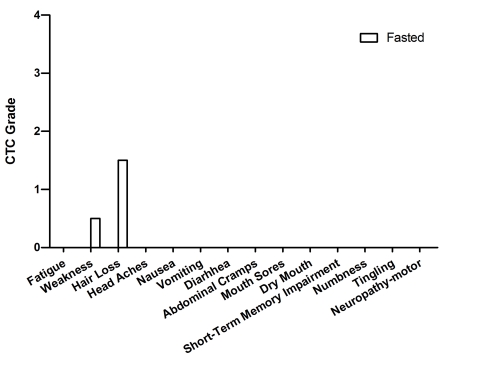
Self-reported side-effects after chemotherapy for case 9. Data represent the average of 4 cycles of chemo-fasting treatments.

This is a 48 year-old Caucasian female diagnosed with breast cancer. Her adjuvant chemotherapy consisted of 4 cycles of doxorubicin (DXR, 110mg/dose) combined with CTX (1100mg/dose) followed by weekly paclitaxel and trastuzumab for 12 weeks. Prior to her first chemotherapy treatment, the patient fasted for 48 hours and reported no adverse effects. During the second and subsequent cycles the patient fasted for 60 hours prior to the chemotherapy followed by 5 hours post drug administration (Table 2). She reported no difficulties in completing the fasting. Although she experienced alopecia and mild weakness, the patient did not suffer from other commonly reported side effects associated with these chemotherapy drugs (Figure 10).

### Case 10

**Figure 11.**
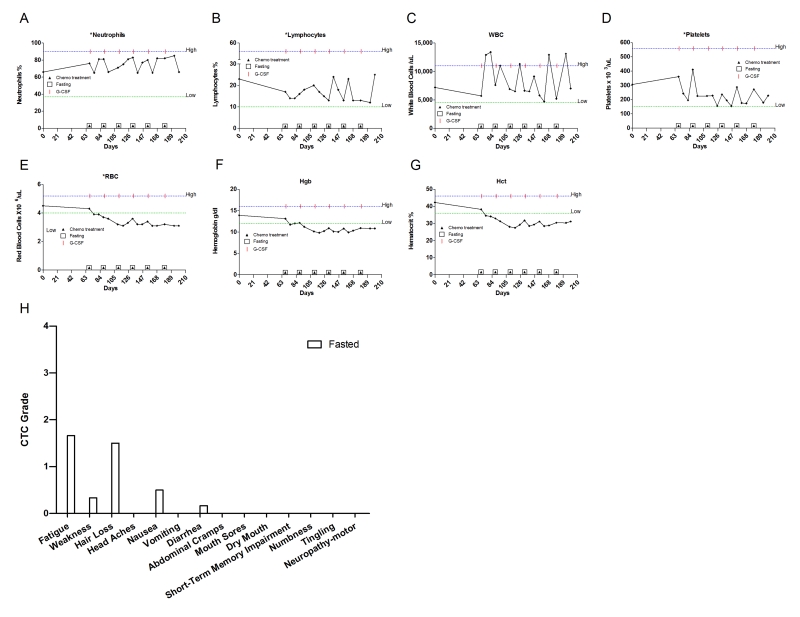
Laboratory values of blood cell counts for case 10. (**A**) Neutrophils; (**B**) Lymphocytes; (**C**) White blood cells, WBC; (**D**) Platelets; (**E**) Red blood cells, RBC (**F**) Hemoglobin, Hgb; (**G**) Hematocrit, Hct. Filled triangle indicates day of chemotherapy; open square indicates fasting. Normal ranges of laboratory values are indicated by dash lines. The patient also received G-CSF (Neulasta) as indicated. (**H**) Self-reported side-effects after chemotherapy for case 10. Data represent the average of 6 cycles of chemo-fasting treatments.

This is a 78 year-old Caucasian female diagnosed with HER2 positive breast cancer. After mastectomy, six cycles of adjuvant chemotherapy were prescribed with CBDCA 400 mg (AUC= 6), TAX (75mg/m^2^) complemented with G-CSF (Neulasta), followed by 6 months of trastuzumab (Table 2). Throughout the treatmen the patient fasted prior and after chemotherapy administration. Although the patient adopted  fasting regimens of variable length, no severe side effects were reported (Figure 11H; Table 2). Her WBC, ANC, platelet and lymphocyte counts remained within normal levels (Figure11A-D) throughout the treatment, but she developed anemia (Figure11E-G).

## Discussion

Dietary recommendations during cancer treatment are based on the prevention or reversal of nutrient deficiencies to preserve lean body mass and minimize nutrition-related side effects, such as decreased appetite, nausea, taste changes, or bowel changes [16]. Consequently, for cancer patients who have been weakened by prior chemotherapy cycles or are emaciated, many oncologists could consider a fasting-based strategy to be potentially harmful.  Nevertheless studies in cell culture and animal models indicate that fasting may actually reduce chemotherapy side effects by selectively protecting normal cells [9]. Following the publication of this pre-clinical work,several patients, diagnosed with a wide variety of cancers, elected to undertake fasting prior to chemotherapy and shared their experiences with us. In this heterogeneous group of men and women fasting was safely repeated in multiple cycles for up to 180 hours prior and/or following chemotherapy. Minor complaints that arose during fasting included dizziness, hunger, and headaches at a level that did not interfere with daily activities. Weight lost during fasting was rapidly recovered in most of the patients and did not lead to any detectable harm.

We obtained self-reported assessments of toxicity from all 10 patients who incorporated fasting with their chemotherapy treatments. Since many of the chemotoxicities are cumulative, we evaluated serial data including all the combined fasting- and non-fasting (*ad libitum* diet) associated chemotherapy cycles (Supplementary Figure 1). Toxicity was graded utilizing a questionnaire based on the Common Terminology Criteria for Adverse Events of National Cancer Institute, version 3.0 (Table 1).  Although the lack of prospective collection of toxicity data and grading are a significant limitation, this series provide an early insight into the feasibility and potential benefit of combining fasting with chemotherapy. Fewer and less severe chemotherapy-induced toxicity was reported by all the patients, even though fasting cycles were often carried out in the later portion of the therapy (Supplementary Figure 1). Nausea, vomiting, diarrhea, abdominal cramps, and mucositis were virtually absent from the reports of all 10 patients in the cycles in which fasting was undertaken prior to and/or following chemotherapy; whereas at least one of these symptoms was reported by 5 out of the 6 patients during cycles in which they ate *ad libitum* (Supplementary Figure 1). The four patients that fasted throughout their treatments reported low severity for the majority of the side effects, in contrast to the typical experience of cancer patients receiving the same chemotherapy regimens (Figures 8I, 9, 10, 11H). For the 6 patients who received chemotherapy with or without fasting, we compared the severity of the self-reported side effects in the 2 closest fasting/non-fasting (*ad libitum diet*) cycles in which the patient received the same chemotherapy drugs at the same dose. There was a general and substantial reduction in the self-reported side effects in combination with fasting (Figure 1). Symptoms such as fatigue and weakness were reported to be significantly reduced (p< 0.001 and p< 0.00193, respectively), whereas vomiting and diarrhea were virtually absent in combination with fasting (Figure 1). In addition, there was no side effect whose average severity was reported to be increased during fasting-chemotherapy cycles (Figure 1 and Supplementary Figure 1**)**.

Challenging conditions such as fasting or severe CR stimulate organisms to suppress growth and reproduction, and divert the energy towards cellular maintenance and repair to maximize the chance of survival [17,19]. In simple organisms such as yeast, resistance to oxidants and chemotherapy drugs can be increased by up to 10-fold in response to fasting/starvetion and up to 1,000-fold in those cells lacking homologs of Ras, AKT and S6 kinase [9]. Nevertheless, such protection and oxidative stress resistance is completely reversed by the expression of oncogene-like genes [9,18]. In mammals, the mechanism(s) responsible for the protective effect of fasting against chemotherapy induced-toxic side effects is not completely understood. It may involve reduction in anabolic and mitogenic hormones and growth factors such as insulin and insuline-like growth factor 1 (IGF-1) as well as up-regulation of several stress resistance proteins[20-25]. In fact, mice with liver specific IGF-I gene-deletion (LID) which have ~80% reduction of circulating IGF-I and mice with genetic disruptions in the IGF-I receptor (heterozygous knockout *IGF-IR* +/-) or its downstream elements have been shown to be more resistant against multiple chemotherapy agents and oxidative stress, respectively [26,27]. Alternatively,  fasting-dependent DSR may be, in part, mediated by cell cycle arrest in normal cells whereas transformed cells continue to proliferate, remaining vulnerable to anticancer drugs [25,28].  Although mutations driving cancer progression are heterogeneous across tumor types, the majority of the oncogenic mutations render cancer cells independent of growth signals [28,29], which we hypothesize prevents cancer cells from responding to the fasting-induced switch to a protected mode [9]. Therefore, DSR would have the potential to be applied independently of the cancer type. Although this has not been yet demonstrated, the remarkable effects of fasting on the down-regulation of a number of growth factors and signal transduction pathways targeted by anti-cancer drugs, including IGF-I and the TOR/S6 kinase pathways, raises the possibility that it could enhance the efficacy of cancer treatment drugs and may even be as effective as some of them.

In summary, in this small and heterogeneous group of cancer patients, fasting was well-tolerated and was associated with a self-reported reduction in multiple chemotherapy-induced side effects. Although bias could affect the estimation of the side effects by the patients, the case reports presented here are in agreement with the results obtained in animal studies and provide preliminary data indicating that fasting is feasible, safe and has the potential to differentially protect normal and cancer cells against chemotherapy in humans. Nevertheless, only a clinical trial, such as the randomized controlled clinical trial currently carried out at the USC Norris Cancer Cen-ter, can establish whether fasting protects normal cells and increases the therapeutic index of chemotherapies.

## Methods

From April 2008 to August 2009, 10 unrelated patients diagnosed with a variety of cancer volunteered to incorporate fasting with their chemo-treatments. We invited these patients to complete a self-assessment survey based on the Common Terminology Criteria for Adverse Events of The National Cancer Institute version 3.0. For the purpose of this study only, we developed a questionnaire that contained 16 easy identifiable and commonly reported side effects; the seriousness of the symptoms was graded from 0 to 4 with each consecutive number corresponding to no side effect/mild/moderate/severe and life threatening. Adverse effects were further divided into 3 major categories including, general, gastrointestinal and central/peripheral nervous system side effects, (Table 1, original questionnaire). The survey was delivered to patients by mail, e mail or fax and every patient was instructed to complete it 7 days after each treatment cycle. Explanation and assistance to patient's concern were offered throughout the study. The eligibility criterion to participate was subjected to those patients that had voluntarily fasted prior and/or post chemotherapy. Medical records including basic demographical information, diagnosis, treatments, imaging studies and laboratory analysis were also retrospectively reviewed (Table 2, Table 3).  All the aforementioned procedures were in compliance with the Internal review Board of the University of Southern California (USC).

## Supplementary material

Supplementary Figure 1 Self-reported side-effects after chemotherapy with or without fasting. Data represent average of CTCAE grade reported by all the patients in this study. 18 chemotherapy cycles under ad-lib diet were compared to 46 chemo-fasting cycles.

Supplementary Table 1 Summary of case 1.

Supplementary Table 2 Summary of case 6. Supplementary material is found at TableS2.docx
