# Supplementary figures and images for: Fasting and cancer treatment in humans: A case series report
Source: Aging (Albany NY). 2009 Dec 31;1(12):988–1007. doi: 10.18632/aging.100114 (PMC2815756; doi:10.18632/aging.100114)

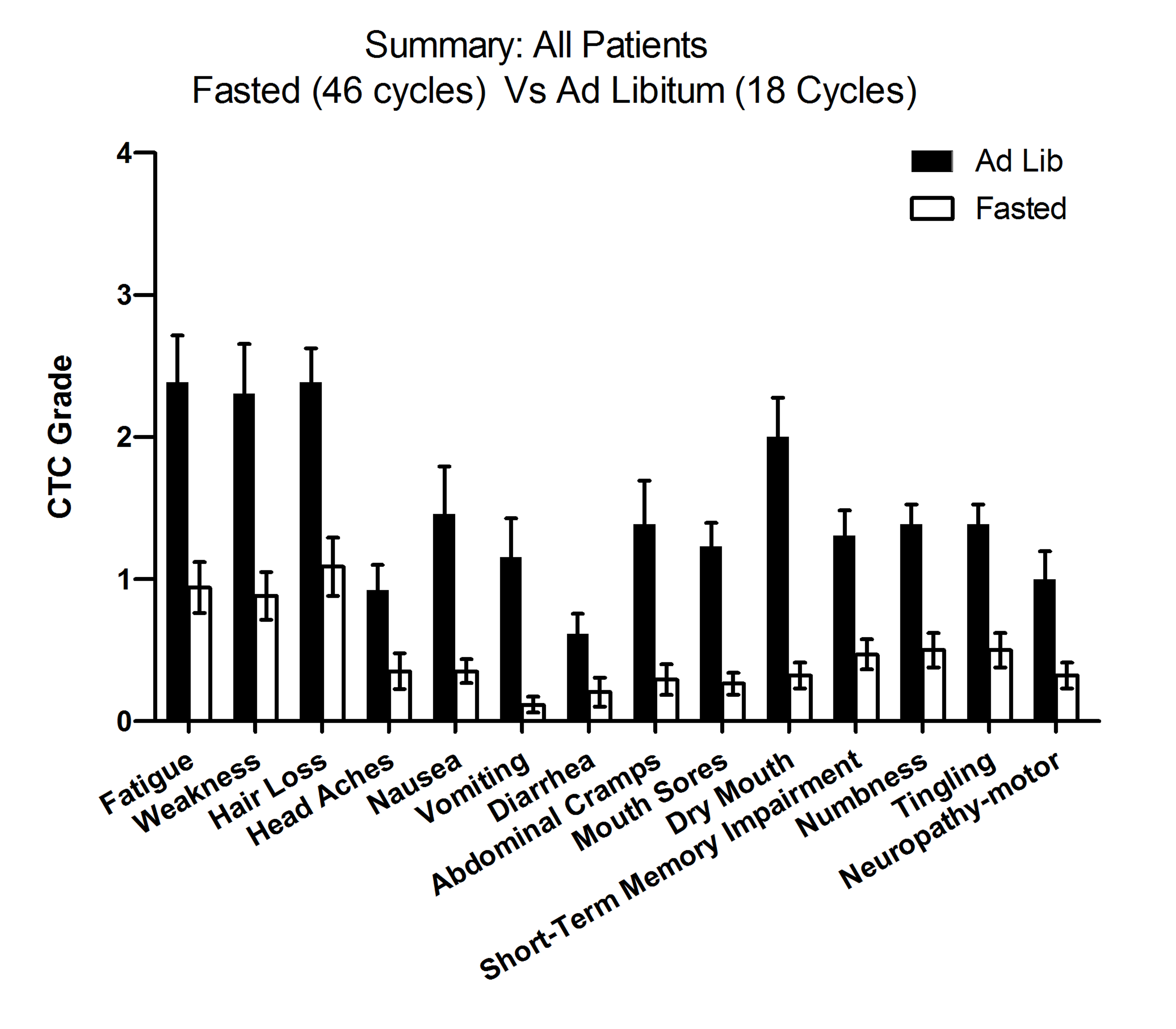

Supplement: Supplementary Figure 1 — Data represent average of CTCAE grade reported by all the patients in this study. 18 chemotherapy cycles under ad-lib diet were compared to 46 chemo-fasting cycles. [file aging-01-988-s001.tif]
