# Supplementary material for: Fasting and cancer treatment in humans: A case series report
Source: Aging (Albany NY). 2009 Dec 31;1(12):988–1007. doi: 10.18632/aging.100114 (PMC2815756; doi:10.18632/aging.100114)
Supplement: Supplementary Table 1 [file aging-01-988-s001.doc]

| **Table S1.**  **Summary of case 1** | | | | |  |  |  |  |  |  |  |
| --- | --- | --- | --- | --- | --- | --- | --- | --- | --- | --- | --- |
| **Days** | **Treatment** | **Fasting (hr)** | | **WBC** | | | | **ANC** | | | |
|  |  | **Pre** | **Post** | **Nadir* (Days)** | **cell/uL** | **Recovery** (Days)** | **Zenith cell/uL** | **Nadir* (Days)** | **cell/uL** | **Recovery** (Days)** | **Zenith cell/uL** |
| 3 | Docetaxel 75mg/m2 + Cyclophosphamide 600mg/m2 | 140 | 40 | **15** | **1700** | **4** | **3900** | **15** | **561** | **4** | **2601** |
| 24 | Docetaxel 75mg/m2 + Cyclophosphamide 600mg/m2 | *ad lib* | **−** | **12** | **1200** | **6** | **4600** | **12** | **120** | **6** | **3036** |
| 45 | Docetaxel 75mg/m2 + Cyclophosphamide 600mg/m2 | *ad lib* | **−** | **12** | **1500** | **8** | **4100** | **12** | **216** | **8** | **2932** |
| 66 | Docetaxel 75mg/m2 + Cyclophosphamide 600mg/m2 | 120 | 24 | **−** | **−** | **−** | **5200** | **−** | **−** | **−** | **3567** |
