# Supplementary material for: Fasting and cancer treatment in humans: A case series report
Source: Aging (Albany NY). 2009 Dec 31;1(12):988–1007. doi: 10.18632/aging.100114 (PMC2815756; doi:10.18632/aging.100114)
Supplement: Supplementary Table 2 — Supplementary material is found at TableS2.docx [file aging-01-988-s002.doc]

| **Days** | **Treatment** | **Fasting (hr)** | |  | **WBC** | | | | **ANC** | | | | **PLT** | | | | |
| --- | --- | --- | --- | --- | --- | --- | --- | --- | --- | --- | --- | --- | --- | --- | --- | --- | --- |
|  |  |  |  | **G-CSF** | **Nadir* (Days)** | **cell/uL** | **Recovery** (Days)** | **Zenith cell/uL** | **Nadir* (Days)** | **cell/uL** | **Recovery** (Days)** | **Zenith cell/uL** | **Nadir* (Days)** | **cell/uL** | **Recovery** (Days)** | | **Zenith cell/uL** |
| 4 | Gemcitabine (900mg/m^2^) | *ad Lib* | **−** | **−** | **7** | **900** | **16** | **9000** | **7** | **400** | **16** | **7500** | **10** | **63** | **11** | **203** | |
| 25 |  |  |  | G-CSF |  |  |  |  |  |  |  |  |  |  |  |  | |
| 27 | Gemcitabine (720mg/m^2^) | *ad Lib* | **−** | **−** | **5** | **700** | **2** | **9200** | **5** | **700** | **2** | **8100** | **9** | **59** | **12** | **177** | |
| 32 |  |  |  | G-CSF |  |  |  |  |  |  |  |  |  |  |  |  | |
| 33 |  |  |  | G-CSF |  |  |  |  |  |  |  |  |  |  |  |  | |
| 34 |  |  |  | G-CSF |  |  |  |  |  |  |  |  |  |  |  |  | |
| 36 |  |  |  | G-CSF |  |  |  |  |  |  |  |  |  |  |  |  | |
| 39 |  |  |  | G-CSF |  |  |  |  |  |  |  |  |  |  |  |  | |
| 41 |  |  |  | G-CSF |  |  |  |  |  |  |  |  |  |  |  |  | |
| 42 |  |  |  | G-CSF |  |  |  |  |  |  |  |  |  |  |  |  | |
| 43 | Gemcitabine (720mg/m^2^) | 62 | 24 | **−** | **5** | **700** | **5** | **7800** | **5** | **700** | **5** | **6400** | **−** |  | **−** |  | |
| 48 |  |  |  | G-CSF |  |  |  |  |  |  |  |  |  |  |  |  | |
| 49 |  |  |  | G-CSF |  |  |  |  |  |  |  |  |  |  |  |  | |
| 50 |  |  |  | G-CSF |  |  |  |  |  |  |  |  |  |  |  |  | |
| 51 |  |  |  | G-CSF |  |  |  |  |  |  |  |  |  |  |  |  | |
| 53 | Gemcitabine 720 mg/m^2^ Docetaxel (80 mg/m^2^) | 62 | 24 | **−** | **4** | **1800** | **3** | **11800** | **4** | **1300** | **3** | **10700** | **8** | **27** | **10** | **280** | |
| 54 |  |  |  | G-CSF |  |  |  |  |  |  |  |  |  |  |  |  | |
| 67 | Gemcitabine (720mg/m^2^) | 62 | 24 | **−** | **9** | **2700** | **5** | **21400** | **9** | **1600** | **2** | **18600** | **−** |  | **−** |  | |
| 76 | Gemcitabine 720 mg/m^2^ Docetaxel (80 mg/m^2^) | 62 | 24 | **−** | **−** |  | **−** |  | **−** |  | **−** |  | **8** | **38** | **6** | **286** | |
| 78 |  |  |  | G-CSF |  |  |  |  |  |  |  |  |  |  |  |  | |
| 91 | Gemcitabine (900mg/m^2^) | 62 | 24 | **−** |  |  |  |  |  |  |  |  |  |  |  |  | |
| 96 |  |  |  | **−** | **6** | **2300** | **1** | **16500** | **6** | **1500** | **1** | **15300** | **−** | **−** | **−** | **−** | |
| 97 |  |  |  | G-CSF |  |  |  |  |  |  |  |  |  |  |  |  | |
| 98 | Gemcitabine (900 mg/m^2^) Docetaxel (100 mg/m^2^) | 62 | 24 | **−** | **6** | **2300** | **1** | **14600** | **6** | **1700** | **1** | **12800** | **7** | **16** | **7** | **250** | |
| 99 |  |  |  | G-CSF |  |  |  |  |  |  |  |  |  |  |  |  | |
| 112 | Gemcitabine (900mg/m^2^) |  |  | **−** |  |  |  |  |  |  |  |  |  |  |  |  | |
